# Second Wave of COVID-19 Global Pandemic and Athletes’ Confinement: Recommendations to Better Manage and Optimize the Modified Lifestyle

**DOI:** 10.3390/ijerph17228385

**Published:** 2020-11-12

**Authors:** Amel Tayech, Mohamed Arbi Mejri, Issam Makhlouf, Ameni Mathlouthi, David G. Behm, Anis Chaouachi

**Affiliations:** 1 Tunisian Research Laboratory “Sport Performance Optimisation”, National Centre of Medicine and Science in Sport (CNMSS), Tunis 1004, Tunisia; ameltayech@gmail.com (A.T.); mejriarbi@gmail.com (M.A.M.); makhloufissam@yahoo.fr (I.M.); ameni.mathlouthy@gmail.com (A.M.); 2 High Institute of Sport and Physical Education, Ksar-Saïd, Manouba University, Tunis 2010, Tunisia; 3 Board Advisor & Debate Trainer, Drabzeen Academy, The International Institute of Debate, Tunis 1002, Tunisia; 4 Faculty of Legal, Political and Social Sciences of Tunis, University of Carthage, Tunis 1054, Tunisia; 5 School of Human Kinetics and Recreation, Memorial University of Newfoundland, St. John’s, NL A1C 5S7, Canada; dbehm@mun.ca; 6 Sports Performance Research Institute, AUT University, Auckland 1010, New Zealand; 7 High Institute of Sport and Physical Education, Sfax University, Sfax 3000, Tunisia

**Keywords:** coronavirus, athletes’ confinement, training camp, performance, detraining, social networks, health, sports nutrition, sleep

## Abstract

Coronavirus 2019 (COVID-19) is an infectious viral disease that has spread globally, resulting in the ongoing pandemic. Currently, there is no vaccine or specific treatment for COVID-19. Preventive measures to reduce the chances of contagion consist mainly of confinement, avoiding crowded places, social distancing, masks, and applying strict personal hygiene as recommended by the World Health Organization (WHO). After the first wave of infection in many countries, the potential effects of relaxing containment and physical distancing control measures suggest that as a result of these measures, a second wave of COVID-19 appears probable in these countries. In sport, the period of self-isolation, and quarantine, for COVID-19 affects the physical preparation of athletes as well as their mental health and quality of life to an even greater extent (i.e., nutrition, sleep, healthy lifestyle), and thus, relevant and practical recommendations are needed to help alleviate these physical and mental health concerns. Our review aims to summarize the physiological and psychological effects of detraining associated with athletes’ confinement during the proposed second wave of COVID-19. This article also proposes answers to questions that concern the advantages and disadvantages of different types of social media platforms, the importance of nutrition, and the effects of sleep disturbance on the health and modified lifestyle of athletes during this worldwide pandemic. Thus, this review provides some general guidelines to better manage their modified lifestyle and optimally maintain their physical and mental fitness with respect to measures taken during this restrictive proposed second wave of the COVID-19 confinement period.

## 1. Introduction

As of 20 September 2020, 30,624,590 people have been confirmed positive for COVID-19 worldwide, with a confirmed mortality of 953,903 [1]. At that date, several countries in the Americas (North and South) are struggling to contain the epidemic, most often due to limited health measures, as in the United States (48,266 new cases), Mexico (4841 new cases), or Brazil (39,797 new cases) [1]. It is suggested that the best prevention is to be well informed about the virus, its causes, and dissemination. From the spread of the pandemic, during the first wave of COVID-19, the WHO [2] advises that people should stay as physically active as possible at home and decrease social interactions. After recording a decline in COVID-19 cases around the world [3,4,5], and after successfully controlling outbreaks early in the year, it has recently been reported that many parts of the world are seeing a resurgence in COVID-19 cases (i.e., a second wave) [6]. Indeed, Leung et al. [7] reported that a second wave of infections would soon follow the premature relaxation of interventions, namely physical distancing control measures during the first-wave COVID-19 transmission. During the confinement period, it is difficult for athletes to perform their routine training that would typically occur with their teammates and under the supervision of coaches and scientific experts. According to the International Olympic Committee (IOC) [8], about 57% of the approximately 11,000 participants in the postponed Olympic Games have already obtained their qualification for the event. However, most of these athletes are in a period of COVID-19 regulation-induced confinement, which may extend into 2021.

Under these conditions of confinement and isolation, athletes are forced to be away from appropriate training conditions and organized competition and have a lack of or restricted communication with coaches. During the more restrictive, limited, and socially isolated training, athletes must be conscious of their safety, maintain stable physical and mental conditions, and be prepared for the resumption of regular training in their clubs. With this in mind, through new media technologies, such as social media platforms [9], several researchers and coaches have formulated specific recommendations and have proposed home training programs to maintain the physical condition of the general population [10], as well as contain, assist, and make the return to practice as easy and secure as possible for all elite and non-elite athletes [11,12,13].

It is important to highlight that appropriate sports nutrition improves performance, reduces fatigue, and limits the risk of illness and injury [14]. It also allows athletes to optimize their training and recover more quickly [15]. Therefore, it is essential to balance energy intake with energy expenditure with the necessary vitamins, minerals, carbohydrates, proteins, fats, and other healthy nutrient constituents. Faced with this radical transformation of daily life, how can the athlete guarantee a healthy and caloric balanced diet during the COVID-19 confinement and physical activity restrictions?

Sleeping well is also essential for athletes’ physical and psychological health. Indeed, sleep is considered necessary not only for optimal performance but also for the physiological and psychological recovery process [16,17]. However, the COVID-19 confinement period can cause sleep disturbances [18,19], which will have an impact on individuals’ daily life, particularly on the athletes’ health [12,13]. Hence, what are the consequences of COVID-19-induced sleep disorders on athletes’ health and how to better manage these sleeping issues during COVID-19 confinement?

Therefore, one objective of this review was to highlight the importance of understanding the physiological and psychological effects of the relative detraining associated with the COVID-19 restrictions, as well as the advantages and disadvantages of different types of social media platforms, the importance of the nutrition, and the effects of sleep disturbance on the health and well-being of athletes. The second purpose of this review is to provide practical recommendations to athletes so that they can optimally maintain their physical and mental conditioning during the COVID-19 restrictions and confinement. With the myriad of athletes and their distinct training needs, it would be impossible to provide particular recommendations fashioned for each athlete. Hence, the recommendations provided within this review are general and focused more on maintaining physical and mental health rather than suggesting highly individualized sport-specific training guidelines.

## 2. Confined Training Methods

The advantage of this critical time (i.e., the second wave of COVID-19) is that we can easily observe and follow the athletes from a distance in their homes. Of course, training on their own can never replace training within a club with a coach and teammates. Against this background, it has been well documented that this modified lifestyle can lead to serious deficits to the quality and quantity of training, with further distancing of the athlete from the reality of their daily training in the traditional preparation sites and uncertainties about the future [20]. The authors emphasize that it is important, during this home confinement, that athletes try to perform the technical movements of their sport, although this is limited in many cases (due to, e.g., dependence on the opponent, such as team sports, need for equipment, or practice location, such as swimming) [20]. In addition to these problems, following home confinement, the interactions and specific tactical behaviors of the players may have decreased the autonomous-stage learning with their teammates within their clubs [20,21]. However, the real constraint is in team (e.g., football, basketball, handball) and dual sports (e.g., tennis, judo, boxing, taekwondo), as they require partners and practice is more effective in game situations. Regarding water sports (e.g., pool, open water, and synchronized swimming, diving, water polo, canoeing, kayaking, rowing, sailing) and ice and snow sports (e.g., skiing, ice hockey, ice climbing, curling, ice skating, speed skating), the situation is more complicated, given that these activities require heavy equipment and specific infrastructures. Therefore, training during home confinement will typically be limited to strength, power, and muscle endurance exercises, general physical preparation (e.g., aerobic training on a cycle ergometer), and stretching, amongst other isolation-limited activities. Acute responses to higher intensities and volumes of exercise can involve a greater risk of illness and impaired immune function [22,23]. In this context, Toresdahl and Asif [11] advise athletes to follow a conservative approach, limiting training sessions to <60 min and to <80% of maximum ability during this time to prevent COVID-19. Herrera-Valenzuela et al. [24] recommended high-intensity interval training (HIIT) for Olympic combat sports athletes that can be performed at home, to maintain their physical fitness, and cardio-respiratory endurance, as well as their musculoskeletal health. In the same line, Jukic et al. [12] warn that detraining is one of the biggest negative consequences of the current “stay at home” confinement. Therefore, the authors recommend that the athlete’s house should be equipped with cardiovascular and resistance training equipment (e.g., portable cycle or rowing ergometer). Athletes must incorporate some type of endurance exercise into their daily routine to avoid the effects of detraining during the forced quarantine. However, to avoid increased chances for upper respiratory tract infections [22,23], the athlete can train at relatively high intensities but should not fully exhaust themselves by the completion of the training session.

The full spectrum of muscle fibers (types I and II: slow and fast-twitch fibers, respectively) should be activated when training [25]. While maximal and near-maximal resistance or maximal explosive actions are often considered necessary to activate the high threshold, type II (fast-twitch) fibers [25], type II fibers can also be recruited when submaximal intensity loads are repeated until task failure or near task failure [26]. Thus, if access to resistance training equipment is limited, the use of body mass exercises such as push-ups [27], chin-ups (pull-ups), lunges, unilateral squats [28], and other exercises can ensure full activation when performed to task failure. Alizadeh et al. [27] performed correlations between push-up and bench press repetitions and found that with each female push-up, it was predicted that they would be able to perform 0.362 bench press repetitions. For each push-up repetition performed by males, it was predicted they could perform 0.973 bench press repetitions. Thus, if 10 standard push-ups (from the toes) are performed, it is predicted that approximately 3–4 and 9–10 bench press repetitions would be performed by women and men, respectively. These predictions would be helpful for athletes in tracking their progress and their eventual return to full equipment availability.

Again, if heavy external resistance equipment (e.g., barbells, dumbbells, machines) is not available, research has shown that higher muscle activation can be achieved with exercises performed under unstable conditions with lower resistance loads or body mass [29,30]. Hence, even without heavy loads, performing body mass squats (bilateral or unilateral), lunges, push-ups, and other exercises using unstable environments like wobble boards, Both Sides Up (BOSU) balls, and sandy surfaces can ensure recruitment of the full spectrum of muscle fibers while also improving balance [31,32,33].

Improving balance, especially in youth athletes, can improve strength, power, and speed performance [33,34,35] and enhance subsequent training adaptations [36,37]. Balance training prior to power (plyometric) training can improve the degree of plyometric training adaptations compared to no prior balance training [37]. Since balance exercises can typically be performed without additional equipment, all athletes and especially young athletes confined during COVID-19 should emphasize balance training exercises at home. Neurodynamic exercises are based on neural mobilization in which force is applied to nerve structures through alterations in posture and multi-joint movement [38] (e.g., tai chi, yoga), which can be incorporated to maintain and improve flexibility and dynamic balance control [12,39,40]. These balance exercises should progress from eyes open to eyes closed, since achieving balance with eyes closed would emphasize additional training adaptations to the vestibular and proprioceptive systems [41]. Furthermore, in accord with the concept of training specificity [42], dynamic (e.g., reverse lunges, unilateral squats, and deadlifts), rather than just static (e.g., standing on one leg) balance exercises, must be practiced. Athletes with greater balance can produce higher force and power outputs [29,30].

Whereas almost all athletes attempt to prepare physically, and technically, do they implement appropriate mental training during this forced quarantine? Jukic et al. [12] reported that this current “stay at home” confinement is an opportunity for both a complete physiological and mental reset as well as for the athlete’s integral development. Although physical activity has a positive effect on athletes’ mental health [43], the adopted home confinement measures and the fear of a second wave of COVID-19, which may delay the return to training and normality and may involve financial problems and depressing daily news, might compromise the athletes’ mental wellbeing while increasing symptoms of anxiety and depression [20]. Indeed, athletes qualified for the Tokyo 2021 Olympic Games must be supported by specialists in the field of mental preparation (i.e., mental imagery) to maintain progress during COVID-19 confinement.

Hence, it is recommended that athletes:

− Maintain their physical activity as effectively as possible [12,20,44,45];
− Take advantage of confinement-induced lack of competitions to emphasize and focus on their weaker physical and mental attributes [12,20,45];
− Use available structures and equipment (e.g., stairs, furniture) to work on balance and resistance training (using body mass and implements to train for strength, power, and endurance). Multiple free videos are available online posted across several social media platforms, including YouTube, Facebook, and Instagram [9]. For an example, see https://www.elevateyourself.org/free-workout-videos.html [46]; https://qz.com/1826582/how-coronavirus-is-making-olympic-athletes-train-under-quarantine [47];
− Train two sessions per day, maintaining three hours between the two sessions. Some athletes may increase the number of daily sessions to try to get around the stress of confinement [20];
− Do not neglect the physical relaxation and stretching exercises after training in order to relax muscles and ensure adequate for super or overcompensation [12,20,45];
− Meditate or use relaxation techniques to reduce anxiety and enhance future performance [48];
− Continue practicing motor simulation and using visualization and mental imagery techniques, which will improve concentration and self-confidence [12,45].

## 3. The Benefits of Social Networks during Athletes’ Confinement

In face of the deserted streets, social networks play a fundamental role during this crisis. Almost 4.57 billion people were active internet users as of July 2020, encompassing 59 percent of the global population [49]. Indeed, the power of social networking is such that the number of worldwide users have dramatically increased during COVID-19, with approximately 3.43 billion monthly active social media users and 3.91 billion monthly active mobile social media users [49]. In 2019, on average, global Internet users spend some 136 min per day surfing social networks. During COVID-19, this number has reached an average of 144 min per day [50]. As of July 2020, Facebook (2.6 billion active users), YouTube (2 billion active users), WhatsApp (2 billion active users), Instagram (1.08 billion active users), Twitter (326 million active users), and others actually play a central role more than ever in social ties [51]. Positive social contacts such as coaches, trainers, and other athletes can encourage athletes to beware of detraining and continue the home training [52,53]. Athletes can virtually connect with their team staff, management, and technical staff, as well as psychologists, physiotherapists, doctors, and sport nutritionists, for online training sessions. Through video conferences, athletes can receive constructive feedback and get the needed support and ensure their progress during this critical period. Indeed, video analysis and feedback are particularly relevant for elite athletes to plan future competitive strategies. Visual and kinesthetic motor imagery, often under the head of “visualization”, is widely used by professional athletes as an effective way to improve motor performance without an overt motor output [54]. Indeed, this cognitive ability allows an athlete to perform and experience motor actions in the mind without actually executing such actions through the activation of muscles [55]. It has been revealed that in some muscles, imagined contractions during visualization training appear to increase strength by inducing purely central nervous system adaptations [56]. Jeannerod [57] reported that the activation of the brain areas needed to perform an action during kinesthetic motor imagery is similar to the activation that occurs during the preparatory planning stages that eventually lead to the action. The author emphasized that kinesthetic motor imagery affects corticospinal excitability. On the other hand, home confinement athletes can use social media to interact with fans, followers, and fellow athletes, build a public image, and present both athletic and personal lives [53]. Finally, these different social media platforms allow athletes to keep in touch with their staff so that they get the necessary motivation and do not feel as isolated [9,58].

Hence, we draw athletes’ attention to the fact that they can:

− Disseminate positive and relevant videos posted across several social media platforms, providing insights into their home workouts, skills training, and challenges against other athletes [9];
− Exploit the power of social media to spread positive messaging and encourage appropriate behaviors [58];
− Promote health messages related to physical activity in online social spaces [9];
− Consult several social media campaigns specializing in COVID-19 confinement athletes training to take advantage of their media coverage [9].

## 4. The Disadvantages of Social Networks on the Mental Health of Athletes during Home Confinement

As media continuously report and often disseminate information related to the numbers of sick and dead to keep everyone informed of the COVID-19 pandemic situation, athletes in total confinement are exposed to this overabundance of information, resulting in negative emotions, less perceived self-efficacy, poor sleep quality, and feelings of loneliness, all associated with higher distress of mental health [20,58]. In this context, sleep deprivation may be a frequent source of stress for athletes during COVID-19 confinement [11,58]. Against this background, it has been well documented that late-night internet addiction is associated with diverse health problems that have been related to delayed sleep onset, and sleep disturbances, that induce negative associations with daytime functioning, persistent fatigue, daytime sleepiness, poor appetite, anxiety, mood alterations, depression, behavioral issues, attention deficit hyperactivity disorder, impaired concentration and performance, caffeine and illicit drug use, increased alcohol consumption, increased metabolic risk and body mass index, and family conflicts [20,59,60,61,62,63].

More research is necessary for psychologists, psychiatrists, and sport sociologists to study the immediate impact of the COVID-19 pandemic on the mental health and the quality of life of athletes, examining levels of psychological impact, anxiety, depression, and stress. Monitoring the individual and collective psychological state of athletes as part of targeted interventions is an absolute necessity during this adapted lifestyle.

We advise athletes to:

− Avoid being over-exposed to the media, which convey anxiety-provoking information. Watch TV once or twice a day only [58];
− Keep in touch with loved ones, family friends, and coaches and scientific experts with the use of different networking and communication technologies available [64];
− Be wary of browsing social media late at night so as not to alter their sleep cycle and circadian physiology [59].

## 5. Nutrition and Lifestyle of Confined Athletes

Nutrition is a key determinant of health [65]. Sports nutrition improves athletic performance because it reduces fatigue and the risk of illness and injury [66]. It also allows athletes to optimize their training and recover faster [15,66]. During confinement, it is essential to balance energy intake and expenditure to prevent an energy deficit or surplus. However, this period is a new situation when boredom and stress cause athletes to lose their usual daily rhythm and incorporate bad habits, such as overeating or snacking, especially of foods rich in sugars, “comfort foods”, fats, and highly processed and readily available foods [67]. In view of the above considerations, Muscogiuri et al. [67] reported that these unhealthy nutritional habits could increase the risk of developing obesity, which is often complicated by heart disease, diabetes, and lung disease, which have been demonstrated to increase the risk for more serious complications of COVID-19. Indeed, dieticians and nutrition experts are invited to educate and provide advice to athletes [12]. In this regard, the WHO [68] recommended the importance of drinking hot drinks and water on a regular basis, especially in this pandemic period. Plan a diet so that athletes can eat four small meals a day, breakfast, lunch, snack, and dinner. Consume daily the essential foods for energy, like rice, pasta, bread, and roots, but in small quantities. Consume daily foods that contribute to physical growth and regeneration of the body: meat, fish, eggs (a good source of low-fat protein and vitamins), milk, and other dairy products such as cheese and yogurt, which are high in tryptophan and zinc. Plant-based protein sources, such as lentils and beans, have a long shelf life and are rich in vitamins and minerals [45]. The consumption of a healthy diet is predominantly vital for the maintenance of immune function, especially important during the COVID-19 confinement [69]. We also encourage the consumption of colored fruits and vegetables (green, yellow-orange, red, and purple) generally rich in vitamins and antioxidants. In this regard, Muscogiuri et al. [67] reported that the antioxidant-rich foods increased the number of T-cell subsets, enhanced lymphocyte response to mitogens, increased interleukin-2 production, potentiated natural killer cell activity, and increased response to the influenza virus vaccine [70]. In addition, canned, frozen, dried, or fermented fruits and vegetables remain an alternative source of vitamins and minerals, in case it becomes difficult to buy fresh products. Avoid foods rich in fats, sugar, and salt as they provide limited nutritional advantage. Finally yet importantly, never neglect vitamin D given its important role in reducing the risk of respiratory tract infections [67,71]. Appropriate levels of vitamin D can be produced endogenously with 10-30 min of midday exposure to the sun each day [72]. While athletes who focus on muscle hypertrophy will have different nutritional needs than, for example, an endurance athlete, the following recommendations emphasize general health recommendations rather than specialized sport-specific needs.

We advise the following:

− Balanced and healthy meals (i.e., appropriate combinations of carbohydrates (55–70% of total energy intake), protein (1.6–2.2 g/kg/day depending on the types of sports and the training phase), and fats (rapeseed oil, olive oil—at least 2 teaspoons per meal) to ensure sufficient energy, vitamins, minerals, and other essential ingredients) [45,67];
− Foods rich in vitamins A, C, E, B6, and B12, zinc, and iron such as citrus fruits, dark green leafy vegetables, nuts, and dairy products [69];
− Avoid eating sugar-rich foods high in saturated fats and instead consume high amounts of fiber, whole grains, unsaturated fats, and antioxidants to boost immune function [73,74];
− Avoid smoking, alcohol, and drugs [69];
− Sufficient hydration. Dehydration increases the risks: athletes should stay well hydrated throughout the day and make sure they are well hydrated before, during, and after the training sessions and do not wear additional clothes to augment the sweat response [45]. The WHO [68] recommends drinking water instead of sugar-sweetened beverages;
− Athletes should drink 2.5 to 3.5 L per day: 500 mL of water in the morning +500 mL between noon and 4 p.m. +500 mL between 4 p.m. and the evening meal +2 glasses of water at each meal +500 mL minimum during training (at least 500 mL but to be adapted according to perspiration). This volume should be increased when it is hot [45];
− Athletes should try to find their rhythm: if they are not hungry, they must not eat. The athletes must attempt to distinguish between hunger and the desire to eat due to boredom or the difficulty of the current situation [45];
− Athletes should limit the loss of their muscle mass and fat intake with resistance training at least twice per week [45].

## 6. Sleep Hygiene and Athletes’ Confinement

As athletes encounter sleep deprivation induced by the COVID-19 confinement stress and related to digital overload, hyper-connection, and digital collaboration, they are challenged to protect their physical and mental health, but some of them may have trouble falling asleep in this major upheaval in life. These following recommendations aim to highlight the importance of the sleep–wake cycle in the field of sport and to clarify for athletes, coaches, and all sports scientists how sleep loss could indirectly compromise the performance of athletes via the indirect alteration of their physical, physiological and psychosomatic process.

According to a recent study [75], National Basketball Association professional athletes have scored fewer points and get fewer rebounds the day after late-night activity on Twitter. In this regard, numerous studies supported the conclusion that partial and/or total sleep deprivation impaired cognitive and physical performance among athletes [16,75]. Indeed, recent findings revealed that one night of partial sleep deprivation decreased the evening resting level of monocytes and mean corpuscular hemoglobin [76] and increased the morning and evening resting levels of the biomarkers of cardiac damage responses (i.e., creatine phosphokinase, ultra-sensitive C-reactive protein, and myoglobin) among taekwondo players [77,78]. Furthermore, it has been well documented among trained athletes that a multitude of biochemical changes within the affected muscle areas such as increased inflammatory cytokines and reactive oxygen species may aggravate muscle injuries and thus increase the cardiac injury risk in response to mainly acute exercise following one night of partial sleep deprivation [78,79]. In addition, psychomotor and cognitive performances were the most affected by sleep deprivation among athletes [80,81]. It has been reported that sleep deprivation can lead to disturbances in the person’s psychological state (for example, increased anxiety, depressed mood, anger, tension, frustration, and irritability) [80] and that any deterioration of this psychological well-being is also likely to affect the performance of the athlete [16]. However, it has been revealed that adequate sleep, both in quantity and quality, is often viewed as a restorative process that influences the homeostatic regulation of the autonomic, neuroendocrine, and immune systems and is generally associated with better physical performances [16].

The following guidelines are proposed to be taken into consideration by athletes during the COVID-19 confinement:

− Adequate sleep is essential to help athletes maintain optimal health and well-being [16];
− Sleep is an important factor in the athlete’s health stress model [12,44,45];
− Athletes must have a good sleep routine (the average sleep duration is between 7 and 8.5 h) by going to bed at the same time in a cool, dark, and quiet room and waking up at the same time [16,59];
− Avoid eating about 3–4 h before sleeping. Going to bed immediately after a large meal can disrupt sleep [59];
− Avoid caffeine or other stimulating drinks about 4–5 h before sleep [16,59];
− Avoid the overuse of social networks (Twitter, Facebook, Instagram) and electronic devices (smartphone, tablet, etc.) in the late night [59].

## 7. Collective Confinement in School or Training Camp as a Suitable Solution during the Second Wave of COVID-19

As another suitable solution during the second wave of COVID-19, we propose that athletes (professional, elite, and student-athletes) would reside in a school or training camp, where the athletes’ nutrition, rhythm of life, sleep, and training program would be rigorously monitored by the administrative and coaching staff [82]. Such a solution could allow coaches to make conscious efforts to maintain the load and intensity of training sessions at the optimum competitive level for these athletes [82]. It has been well established that exercise intensity is the key factor that influences the quality of effort and thus the overall efficacy of athletic training programs in this collective confinement model compared to athletes who are in self-confinement [83]. There appear to be good reasons to apply a similar model (e.g., collective confinement in school or training camp) in developing plans to avoid detraining associated with self-confinement of athletes during the second wave of COVID-19. Indeed, the physiological systems that underpin performance can be affected by many external factors, such as nutrition status [12,65,67], sleep [12,18,19], stress and anxiety of self-confinement, mood alterations, and depression [58]. Accordingly, any change in these patterns, such as occurs during COVID-19, has the potential to reduce the physical performance of self-confined athletes [12,20,44,45]. However, such conditions (i.e., collective confinement in school or training camp) are clearly not always readily available to many teams and athletes.

## 8. Conclusions

Athletes’ lives are disrupted during the COVID-19 pandemic. There is a major psychological impact of athletes’ confinement due to this modified lifestyle because they have no reference to this new situation. It is evident that athletes must follow a balanced, healthy lifestyle, namel, exercise, nutrition, rhythm of life, and getting enough sleep. However, it is difficult to perform routine training within the context of the ongoing pandemic and confinement. Most athletes will find themselves confronted with the search for an optimal solution to maintain their physical, physiological, and psychological levels as close as possible to their original skills. Thus, this review serves as a general guideline mainly for athletes to optimally maintain being physically and mentally fit with respect to measures taken in the face of COVID-19 confinement period with recommended guidelines. If an athlete’s situation changes, allowing for more intensive and extensive training, then they can hopefully return to more sport-specific recommendations for training, nutrition, and other training components. It is important to mention that this article does not concern athletes only, but it also raises a call for the sports team to act proactively and keep ties with their trainees and provide them with the needed guidance and motivation through the best use of social media platforms. Therefore, everyone will have to adapt to different situations. Obviously, everything is based and depends on the level of consciousness of the athlete himself.
