# Correction: Tayech, A., et al. Second Wave of COVID-19 Global Pandemic and Athletes’ Confinement: Recommendations to Better Manage and Optimize the Modified Lifestyle. *Int. J. Environ. Res. Public Health* 2020, *17*, 8385

**DOI:** 10.3390/ijerph18062850

**Published:** 2021-03-11

**Authors:** Amel Tayech, Mohamed Arbi Mejri, Issam Makhlouf, Ameni Mathlouthi, David G. Behm, Anis Chaouachi

**Affiliations:** 1 Tunisian Research Laboratory “Sport Performance Optimisation”, National Centre of Medicine and Science in Sport (CNMSS), Tunis 1004, Tunisia; ameltayech@gmail.com (A.T.); mejriarbi@gmail.com (M.A.M.); makhloufissam@yahoo.fr (I.M.); ameni.mathlouthy@gmail.com (A.M.); 2 High Institute of Sport and Physical Education, Ksar-Saïd, Manouba University, Tunis 2010, Tunisia; 3 Board Advisor & Debate Trainer, Drabzeen Academy, The International Institute of Debate, Tunis 1002, Tunisia; 4 Faculty of Legal, Political and Social Sciences of Tunis, University of Carthage, Tunis 1054, Tunisia; 5 School of Human Kinetics and Recreation, Memorial University of Newfoundland, St. John’s, NL A1C 5S7, Canada; dbehm@mun.ca; 6 Sports Performance Research Institute, AUT University, Auckland 1010, New Zealand; 7 High Institute of Sport and Physical Education, Sfax University, Sfax 3000, Tunisia

In our recently published article [1], the reference [2] “Chandra, R.K. Effect of vitamin and trace-element supplementation on immune responses and infection in elderly subjects. Lancet 1992, 340, 1124–1127.” has been retracted.

Therefore, we sincerely apologize for missing this important item and we have eliminated the Chandra’s reference [2] from our review and replaced it with the Thurnham’s reference [3] to support the statement about the benefits of the antioxidant-rich foods to strengthen immunity. We state that the scientific conclusions are unaffected. The original article has been updated.

We apologize for this mistake and appreciate the diligence of Jiménez-Gómez and colleagues in spotting this error and helping to maintain the integrity of the science in this field.
